# Radiological and Surgical Findings of Patients with Intradiploic Mass Lesion: Case Series and Systematic Review

**DOI:** 10.1055/a-2731-4781

**Published:** 2026-06-26

**Authors:** Abdulkerim Gökoğlu, Hüseyin Yiğit, Merdan Orunoğlu, Turgut Tursem Tokmak, Ayça Erşen Danyeli, Erdoğan Unur, Ahmet Selçuklu

**Affiliations:** 1 Department of Neurosurgery, Private System Hospital, Kayseri, Turkey; 2 Department of Medical Services and Techniques, Cappadocia University, Nevşehir, Turkey; 3 Department of Neurosurgery, Kayseri State Hospital, Kayseri, Turkey; 4 Department of Radiology, Kayseri Research and Training Hospital, Kayseri, Turkey; 5 Department of Pathology, Acıbadem MMA University, School of Medicine, Istanbul, Turkey; 6 Department of Anatomy, Faculty of Medicine, Erciyes University, Kayseri, Turkey; 7 Department of Neurosurgery, Erciyes University, School of Medicine, Kayseri, Turkey

**Keywords:** intradiploic mass, epidermoid cyst, MRI, CT

## Abstract

**Background and Objectives:**

In this study, our goal is to examine the radiological findings and clinical outcomes of nine patients with intradiploic masses, who were treated at our clinic, along with a comprehensive review of the existing literature.

**Methods:**

The study includes a total of nine adult patients, who were under follow-up and treatment for intradiploic masses from 2015 to 2022. Exclusions from the study criteria comprised patients in the pediatric age group, those with a documented history of cancer, prior cranial surgery, active central nervous system infection, and acute head trauma resulting in cranial damage.

**Results:**

Our study comprised a total of nine patients, with six females and three males, with a median age of 36 years (range: 18–76). Epidermoid cysts were identified in two patients, whereas others presented with cavernous hemangioma, arachnoid/leptomeningeal cyst, intradiploic lipoma, dermoid cyst, arachnoid cyst, fibrous dysplasia, and eosinophilic granuloma. Among the cohort, eight patients presented with headaches. The patient with cavernous hemangioma underwent total resection and mini-plate reconstruction. Similarly, total resection was performed in cases of leptomeningeal cyst ( *n* = 1) and intradiploic lipoma ( *n* = 1). In a single patient, fibrous dysplasia was diagnosed through open biopsy. For the patient with eosinophilic granuloma, total mass excision and chemotherapy were undertaken. Notably, four patients (44.4%), including those with epidermoid cysts ( *n* = 2), dermoid cyst ( *n* = 1), and arachnoid cyst ( *n* = 1), were managed conservatively without surgical intervention.

**Conclusions:**

The use of computed tomography and magnetic resonance imaging in intradiploic lesions seems sufficient to differentiate the mass. However, it may be difficult to reach a definitive diagnosis in some patients without surgery. Therefore, based on the experience of clinical management, it is important to evaluate in detail the various radiological and clinical findings for further therapeutic decision making.,

## Introduction

Intradiploic lesions can originate from bony structures or result from the invasion of the skull by scalp or brain lesions. Typically asymptomatic, calvarial lesions are often discovered incidentally during brain computed tomography (CT) or magnetic resonance imaging (MRI), or when evaluating local clinical symptoms or other diseases. 1 2 3 4 5 6 In some cases, these lesions may become symptomatic by visible or palpable swelling. 1 4 Key clinical factors, such as age and medical history, play a crucial role in guiding the radiological diagnosis. While calvarial lesions can be either benign or malignant, the majority are benign tumors. 1 2 3 4 5 6

Recognition of benign and malignant behavior imaging features is important for diagnosis. In general, benign tumors have well-defined borders with a narrow transition zone; sclerotic margins are frequently present. However, malignant tumors have poorly defined margins, a wide transition zone, aggressive periosteal reaction, and often have a soft tissue component; these lesions can cause dramatic bony destruction with intracranial or extracranial extension. Skull lesions can be lytic or sclerotic, single or multiple with varied composition. 7

The current study focuses on intradiploic lesions, aiming to elucidate their distinct features and clinical significance, thereby differentiating them from intradural pathologies affecting the brain and spine. The majority of these tumors are meningiomas and schwannomas, comprising 80%, whereas filum terminale ependymomas account for around 15%. Conversely, extradural tumors constitute approximately 55% of spinal masses and are predominantly metastatic and malignant. 8 These tumors either remain asymptomatic or are incidentally detected or they may present as a palpable lump. They can erode the bone and involve the brain parenchyma due to their proximity to the brain. Radiological imaging is very helpful in accurate diagnosis of these lesions and in differentiating intradural from intradiploic lesions. 9 MRI is the most effective modality for assessing marrow involvement within the diploe as well as evaluating associated soft tissue components and the invasion of nearby structures. 10

This study focuses on reviewing the radiological findings and clinical outcomes of nine patients with intradiploic masses, who were treated at our clinic, accompanied by a comprehensive literature review.

## Materials and Methods

### Clinical Case Selection

Patients diagnosed with intradiploic masses followed in our clinic were included in this study. Verbal and written consent was obtained from all patients participating in the study. The complaints of all patients were recorded in detail. Detailed physical examination and neurological tests were performed. CT and MRI scans of the patients were interpreted by two separate radiologists, each 10 years of experience in neuroradiological imaging, blinded to the study.

All patients received treatment for intradiploic masses at our clinic from 2015 to 2022. Every patient included in the study provided a signed informed consent form.

The following patients were excluded from the study: children, those with a documented history of cancer, those with prior cranial surgery, those with an active central nervous system infection, and those with acute head trauma resulting in cranial damage.

### Surgery

In the absence of vascular disorders, compression of neural tissues, or compromise of tissue integrity, conservative symptomatic treatment is generally preferred. Surgical intervention was considered for lytic lesions or lesions with expansile mass effects.

### Systematic Literature Review

A systematic search was undertaken in PubMed and Embase databases from inception to May 10, 2024. Medical Subject Headings (MeSH) used for the search were as follows: “intradiploic masss” OR “epidermoid cyst” OR “dermoid cyst” AND (“MRI” OR “CT”). The following keywords were used with the MeSH terms: “intradiploic masss,” (“epidermoid cyst, dermoid cyst, skull, eosinophilic granuloma, headache, arachnoid cyst, malignant epidermoid”). Titles and abstracts identified were screened by both authors. A manual search of secondary sources included personal holdings, conference abstracts, and review of the references of identified articles. Graph 1 shows the study flow as recommended by the PRISMA 2020 updated guideline. 11

**Graphic 1:**
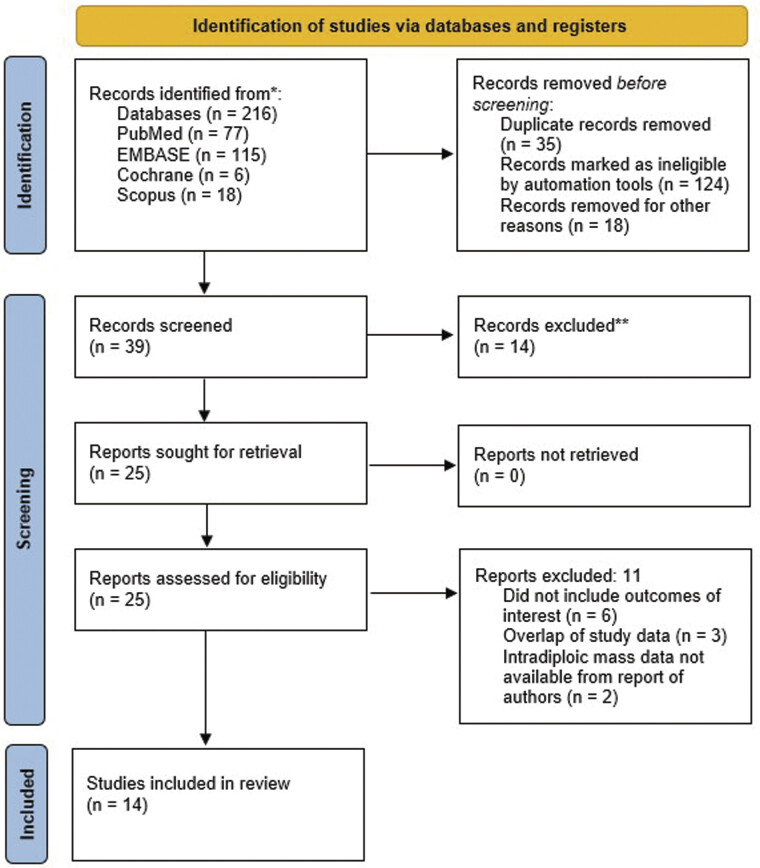
PRISMA flow diagram for systematic literature review.

### Screening and Data Extraction

Two authors trained in performing systematic reviews and database searching conducted the search strategy. In the first stage, the titles and abstracts of the articles were reviewed; in the next stage, two authors independently reviewed the full text of the articles. After preparing a list of titles and summaries of studies, articles were evaluated in terms of various methodological aspects, including sampling methods, reliability of the tools used, and the objectives of the study. In case of confusion due to controversial literature findings, it was planned to include the studies in the research by reaching an expert consensus with the third independent author. Finally, a set of articles appropriate in terms of topic coverage and content structure was included in the article.

## Results

### Clinical Characteristics

Our study included a total of nine patients (66.6% females and 33.3% males) with a median age of 36 years (range: 18–76). The patients presented with various conditions, such as epidermoid cyst in two cases. Other cases included cavernous hemangioma, arachnoid/leptomeningeal cyst, intradiploic lipoma, dermoid cyst, arachnoid cyst, fibrous dysplasia, and eosinophilic granuloma. Table 1 provides a summary of the clinical and radiological findings. Headache was a presenting symptom in eight patients. Notably, the patient with cavernous hemangioma experienced a speech disorder, the patient with fibrous dysplasia had left eye proptosis, the patient with an arachnoid cyst presented with right parietal swelling, and the patient with an intradiploic lipoma had palpable swelling adjacent to the sagittal suture. MRI was available for eight patients. The diagnosis was confirmed by CT in one case. Patient-based clinical characteristics and treatment outcomes are summarized below ( Table 1 ).

**Table 1:** Summary of the clinical and radiological findings of the patients

Patient number	Age	Gender	Complaints and findings	Imaging	Preoperative image	Postoperative image	Localization	Procedure performed	Diagnosis
1	25	Female	Speech disorder, right lateral weakness, effaced left nasolabial fold , DTR hypoactive	T1 and T2 DWI hyperintense	Y	N	Left frontal	Total resection, mini-plate reconstruction	Cavernous hemangioma
2	33	Female	Headache, right parietal swelling, history of traffic accident	T1 DWI hypointense, T2 DWI hyperintense	Y	Y	Right parietal and right parasagittal	Cystectomy, total resection	Leptomeningeal cyst
3	26	Female	Headache, palpable soft swelling in the midline of the head, oval, painful, soft mass fixed to the skull at the vertex	T1 DWI homogeneous, T2 and FLAIR hyperintense	Y	Y	Right parietal and right parasagittal	Total resection	Intradiploic lipoma
4	76	Female	Headache	T1 hypointense and T2 FLAIR hyperintense	Y	N	Right parietal and right parasagittal	Nonsurgical follow-up	Epidermoid cyst
5	36	Male	Headache, swelling in the frontal area	T1 hypointense and T2 hyperintense discrete contrast enhancement in the periphery	Y	N	Left frontal	Nonsurgical follow-up	Epidermoid cyst
6	72	Female	Headache	T1 hyperintense, T2 isointense, DWI isointense	Y	Y	Right frontal	Nonsurgical follow-up	Dermoid cyst
7	70	Male	Headache	T1 DWI hypointense, T2 DAG hyperintense, T1 with contrast nonenhancing	Y	N	Left occipital	Nonsurgical follow-up	Arachnoid cyst
8	46	Female	Proptosis of the left eye, headache, skull deformity around the left eye	Intradiploic lesion causing destruction of the left parietal and zygomatic bone on axial cranial CT scan	Y	Y	Left orbital and zygomatic	Biopsy	Fibrous dysplasia
9	18	Male	Persistent headache after trauma	T2 and FLAIR hyperintense and T1 isointense	Y	Y	Right parietal bone	Excision	Eosinophilic granuloma

Abbreviations: CT, computed tomography; DWI, diffusion-weighted imaging; FLAIR, fluid-attenuated inversion recovery; MR, magnetic resonance; N, no; Y, yes;

#### Case 1, cavernous hemangioma

**Fig. 1:**
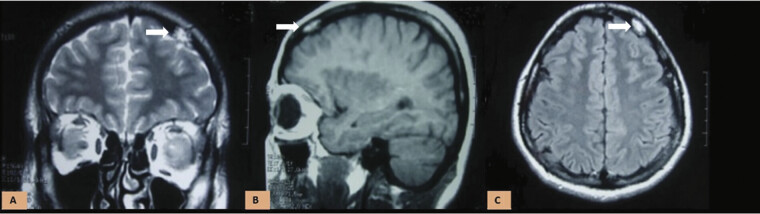
Coronal T2-weighted ( **A** ), sagittal T1-weighted ( **B** ), and axial FLAIR ( **C** ) images reveal a well-defined prominent hyperintense lesion measuring 20 × 8 × 20 mm, located in the intradiploic region of the left frontal bone. The radiologic features are consistent with an intradiploic cavernous haemangioma, as confirmed by histopathological analysis. MR, magnetic resonance.

A 25-year old woman presented to our outpatient clinic with a speech disorder and weakness on the right side. On physical examination, there was a slight effacement in left nasolabial sulcus and hypoactive deep tendon reflexes in upper extremity. On cerebral MRI, an intradiploic cystic lesion (20 × 8 × 20 mm) was observed in the left frontal skull region, which showed hyperintensity on T1- and T2-weighted images ( Fig. 1A–C ). The patient underwent total resection of the lesion with mini-plate reconstruction. Neuronavigation was employed to delineate the surgical target and margins. A curvilinear skin incision was made to access the lesion. Using a high-speed drill, the bony margins were circumferentially addressed, and the lesion was completely excised. Histopathology was cavernous hemangioma. The patient experienced an uneventful postoperative course.

#### Case 2, arachnoid/leptomeningeal cyst

**Fig. 2:**
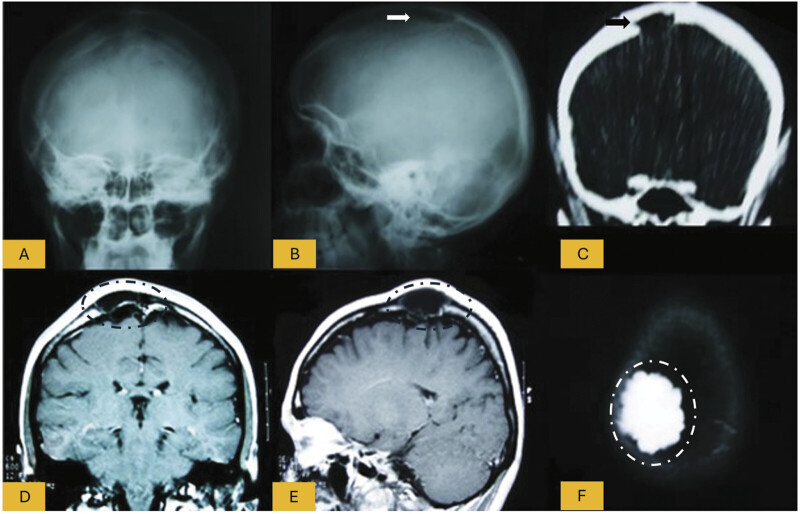
AP and lateral skull radiographies show a well-defined expansile lytic lesion located in the intradiploic region of the right parietal bone adjacent to the vertex region (white arrow) ( **A** , **B** ) A coronal CT scan was performed after the surgical removal of the lesion, which shows a bone defect repaired with a cranioplasty procedure using a synthetic porous polyethene graft (black arrow) ( **C** ). Coronal and sagittal T1-weighted post contrast images (black circle) ( **D** , **E** ) demonstrate hypointense expansile cystic lesion with no contrast enhancement, the lesion is seen prominent hyperintense signal on the axial T2-weighted image (white circle) ( **F** ) The radiologic features are consistent with a leptomeningeal cyst, as confirmed by histopathology. AP, anterior -posterior; CT, computed tomography; MR, magnetic resonance.

A 32-year old woman presented with chronic headache and swelling (2 cm in size initially and reached up to 4 cm after 2 months) in the right parietal region. She had a history of head trauma due to a motor vehicle accident nearly 4.5 years ago. In skull radiographs, a wide radiolucent area with sclerotic margins was observed at right parietal region ( Fig. 2A, B ). On cranial postoperative coronal CT scan, there was internal table defect due to enlarged diploic space and thinned external table ( Fig. 2C ). On cranial MRI a round, cystic mass filled with cerebrospinal fluid (CSF) was observed in axial sections, which appeared hypointense on T1-weighted images and hyperintense on T2-weighted images ( Fig. 2D–F ). Following a modified curvilinear skin incision, a skin flap was elevated, providing access to the lesion. Due to the lesion's gross size, neuronavigation was deemed unnecessary. During surgery, extremely thinned external table was observed when scalp flap was removed. Cystic contents were drained during the craniotomy, exposing a thin membrane surrounding the cyst. Total resection was performed. Following resection of the mass, the resulting bony defect was repaired using porous polyethylene. An optimal cranioplasty was achieved using synthetic grafting contoured to the cranial convexity ( Fig. 2C ). Histopathological examination showed an arachnoid/leptomeningeal cyst. There were no complications.

#### Case 3, lipoma

**Fig. 3:**
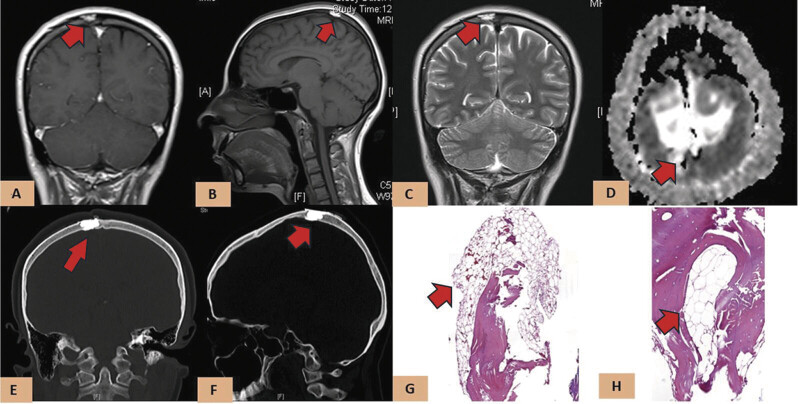
Coronal and sagittal T1-weighted images, as well as a coronal T2-weighted image, reveal a hyperintense lesion compressing the superior sagittal sinus. The lesion measures 26 × 12 × 20 mm. ( **A** , **B** , **C** ). The axial ADC image reveals no diffusion restriction ( **D** ). Postoperative coronal and sagittal CT images show cranioplasty with polymethyl methacrylate ( **E** , **F** ). Lipid vacuoles are visible at the centre of the histopathological staining ( **G** and **H** , red arrows). CT, Computed tomography; MR, Magnetic resonance.

A 26-year old woman presented with headache and a smooth swelling palpable at the midline of her head. On physical examination an oval, painful, soft mass was detected (3 cm in diameter) fixed to the skull at the vertex. Neurological examination was normal. On cerebral CT scan, there was a defect in the tabula interna with enlarged diploic space and complete destruction of tabula externa. In the diploic space, there was a hyperintense lesion in the right parasagittal region on coronal T1-weighted magnetic resonance (MR) images ( Fig. 3A ). A cystic mass was identified slightly compressing superior sagittal sinus on a T1-weighted MR image ( Fig. 3B ). A hyperintense lesion was noted in the right parasagittal region invading the external and internal tables of the calvaria on coronal T2-weighted MR images ( Fig. 3C ). Axial images showed a round, lobulated, cystic lesion. The lesion was surgically removed. The patient was positioned in a Mayfield skull clamp with the head flexed 15 degrees. Neuronavigational planning was utilized to define the lesion and surgical margins. A midline parasagittal incision, centered over the lesion, was performed. Hemostasis was achieved and retractors were placed. Using a high-speed drill, the bony resection was carefully completed circumferentially around the lesion. Cranioplasty was performed by repairing the defect with acrylic synthetic cement. Lipid vacuoles were seen at the center on histopathological staining ( Fig. 3G and H ). Histopathological examination showed an intradiploic lipoma. Postoperatively, all the symptoms resolved. No adverse events were observed.

#### Case 4, epidermoid cyst

**Fig. 4:**
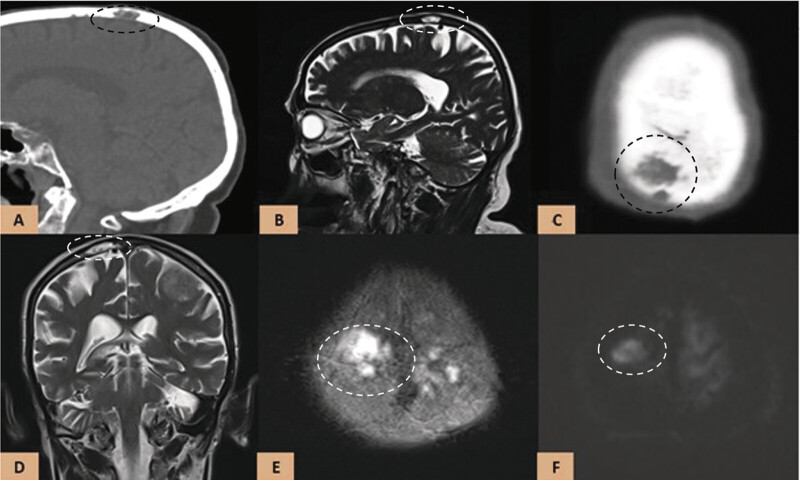
Right parasagittal epidermoid cyst. A cystic hypodense mass lesion (18 × 9 × 13 mm) compressing the superior sagittal sinus sagittal CT image ( **A** ). A hyperintense cystic mass lesion compressing the superiorsagittal sinus on sagittal T2-weighted MR images ( **B** ). A hypodense cystic lesion on axial CT image ( **C** ). A hyperintense lesion and a left frontal meningioma on coronal T2-weighted MR images ( **D** ). A hyperintense, cystic lesion on axial T2-weighted MR images ( **E** ). Restricted diffusion is noted on the diffusion-weighted image ( **F** ). CT, computed tomography; MR, magnetic resonance.

A 76-year-old patient presented with headache. Neurological examination was normal. There was no palpable lesion in physical examination. CT and other imaging findings suggested an intradiploic epidermoid tumor located in the patient's right parietal region. However, radiological images did not reveal mass effect, which could directly explain the patient's headache or lead to periosteal irritation. The location of the tumor could not result in increased intracranial pressure. Fundoscopic examination was normal. There was no neurological motor deficit. Therefore, the patient was placed under observation. In addition, a meningioma was detected in left parietal region. No surgery was performed as the patient had no clinical symptoms. A cystic mass lesion compressing the superior sagittal sinus was seen on T1-weighted MR images ( Fig. 4A ). The cystic mass compressed the superior sagittal sinus on T2-weighted MR images ( Fig. 4B ). A hypodense cystic lesion was seen in the right parasagittal region on axial CT ( Fig. 4C ). A hyperintense, intradiploic lesion was seen in the right parasagittal region and a meningioma was seen in the left frontal region on coronal T2-weighted MR images ( Fig. 4D-F ). No growth or progression of the mass was observed during follow-up. Throughout the patient's follow-up period, no adverse events were observed.

#### Case 5, epidermoid cyst

**Fig. 5:**
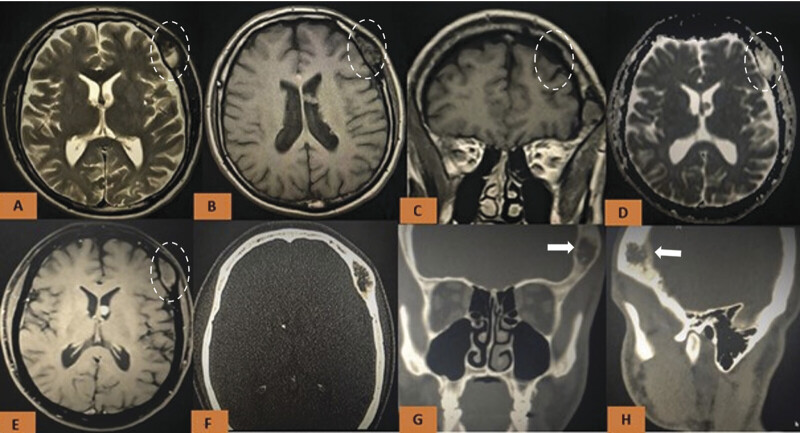
Left frontal epidermoid cyst. A hyperintense, intradiploic cystic lesion (41 × 26 × 22 mm) on axial T2-weighted MR images ( **A** ). An isohypointense, mass lesion on axial T1-weighted MR images ( **B** ). An isohypointense cystic mass lesion on coronal T1-weighted MR images ( **C** ). An axial ADC image shows mild-to-moderate diffusion restriction ( **D** ). On the axial post-contrast T1-weighted image, an expansile hypointense lesion is noted in the left frontoparietal region. Additionally, the image demonstrates a contrast-enhanced intraventricular lesion consistent with a subependymal giant cell astrocytoma in the frontal horn of the left lateral ventricle ( **E** ) **.** Axial, coronal and sagittal CT images reveal an expansile, intradiploic, hypodense lesion in the left frontoparietal region ( **F** , **G** , **H** ). The radiological features are consistent with an epidermoid cyst. CT, computed tomography; MR, magnetic resonance.

A 36-year-old man presented with headache and a dysmorphic head appearance. The patient had history of tuberous sclerosis. The neurological examination was found to be normal. In physical examination, there was skull deformation including swelling at frontal region of skull. Laboratory tests revealed a vitamin D deficiency. In addition, the patient had pyoderma. A cystic lesion was detected in left frontal region on paranasal CT scan and cranial MRI. The lesion was interpreted as epidermoid cyst. Moreover, the patient had a subependymal giant cell astrocytoma in frontal horn of left lateral ventricle. An axial T2-weighted MR image demonstrated a mixed intradiploic mass in the left frontal region, with hypointense anterior portions and predominantly hyperintense components ( Fig. 5A ). An iso-hypointense intradiploic cystic mass lesion was noted in the left frontal region on axial T1-weighted MR images ( Fig. 5B ). An intradiploic, iso-hypointense cystic mass was seen in the left frontal region on coronal T1-weighted MR images ( Fig. 5C ). An intradiploic mass was seen in the left frontal region on axial ADC, contrast-enhanced MR images ( Fig. 5D ). The patient expressed fear of surgery and declined to undergo the procedure. The patient exhibited no neurological deterioration. The patient was advised to seek surgical intervention should any neurological symptoms, hemorrhage, or infection develop. No progression of the mass was observed during a 2-year follow-up period.

#### Case 6, dermoid cyst

**Fig. 6:**
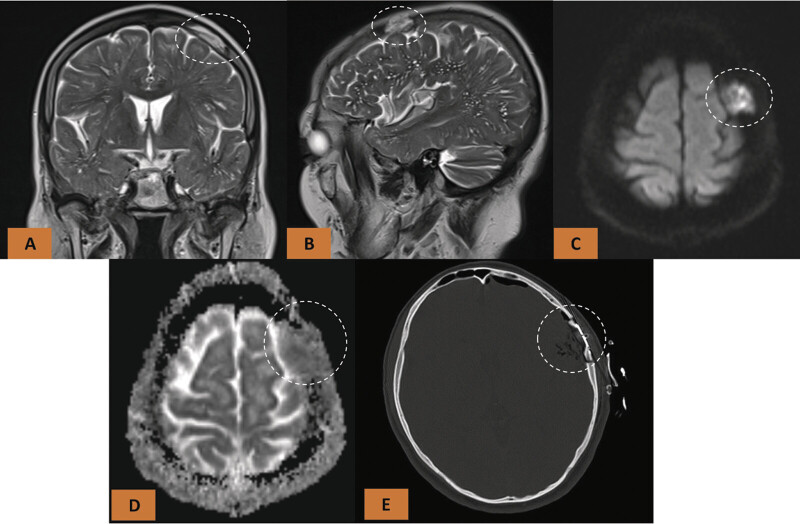
Left frontal dermoid cyst. A non-expansile iso/hyperintense cystic lesion is noted on coronal and sagittal T2-weighted MR images ( **A** , **B** ). The lesion reveals restricted diffusion on the axial ADC-diffusion weighted MR images ( **C** , **D** ). Postoperative axial CT image shows cranioplasty with titanium mini-plates and mini-screws following the complete resection of the lesion ( **E** ). The lesion was found to be fat-enriched filled with intense hair follicles. The radiologic and gross pathologic features are consistent with a dermoid cyst, as confirmed by histopathological analysis. CT, computed tomography; MR, magnetic resonance.

A 72-year old woman presented with headache. The neurological and physical examination was normal. On coronal MRI, an isointense left frontal lesion, suggestive of a dermoid cyst with intense hair follicles was seen ( Fig. 6A ). The lesion compressed superior sagittal sinus on T2-weighted MR images ( Fig. 6B ). A hyperdense cystic lesion was seen in the right frontal region on axial T1-weighted MR images ( Fig. 6C ). An intradiploic, isointense lesion was seen in the right frontal region on axial diffusion-weighted MRI ( Fig. 6D ). Neuronavigation was used for planning the operation. A curvilinear skin incision was made, and the skin flap was deflected. The tumor was totally resected with a high-speed drill. The lesion was found to be fat-enriched. The procedure was completed with cranioplasty using titanium micro-crews and plates. Postoperative axial CT imaging revealed total resection of the dermoid cyst and cranioplasty with titanium mini-plates and mini-screws ( Fig. 6E ).The pathologic investigation showed a dermoid cyst. The postoperative period and follow-ups were uneventful.

#### Case 7, arachnoid cyst

**Fig. 7:**
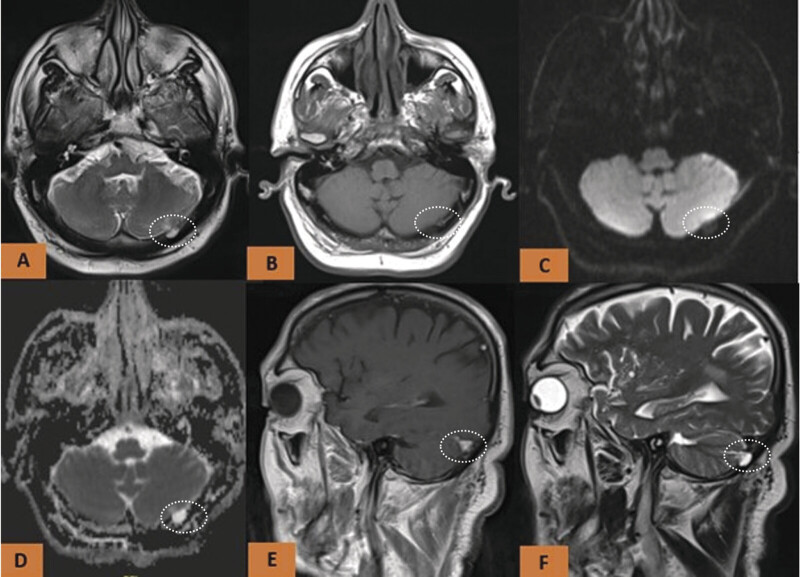
An intradiploic left occipital arachnoid cyst (a non-expansile cystic mass lesion measuring 10 × 6 × 7 mm) shows a hyperintense signal on the axial T2-weighted image and a hypointense signal on the axial T1-weighted MR image (white circles) ( **A** , **B** ). No restricted diffusion is noted on the axial ADC-diffusion weighted MR images (white circle) ( **C** , **D** ). The lesion shows no contrast enhancement on the sagittal post-contrast T1-weighted image, and shows a prominent hyperintense signal on the sagittal T2-weighted image (white circle) ( **E** , **F** ). MR: magnetic resonance.

A 70-year-old male patient complained headache and occipital soft swelling. The neurological and physical examination was normal. Axial T2-weighted MR images revealed a hyperintense cystic lesion in the left occipital diploe, without diffusion restriction and gadolinium contrast enhancement, suggestive of an arachnoid cyst ( Fig. 7A ). On axial T1-weighted MR images, an intradiploic, hypointense cystic mass was seen ( Fig. 7B ). On axial ACD-diffusion T2-weighted MR images, the lesion was hypointense cystic ( Fig. 7C ). On axial diffusion-weighted MR images, an intradiploic, hyperintense lesion without diffusion restriction was noted in the left occipital region ( Fig. 7D ). On contrast-enhanced sagittal T1-weighted MR images, the lesion was hyperintense and not contrast enhancing ( Fig. 7E ). On sagittal T2-weighted MR images, an intradiploic arachnoid cyst was seen in the left occipital region ( Fig. 7F ). No enlargement of the cyst size was observed during the follow-up. The patient's headaches resolved with medical treatment.

#### Case 8, fibrous dysplasia

**Fig. 8:**
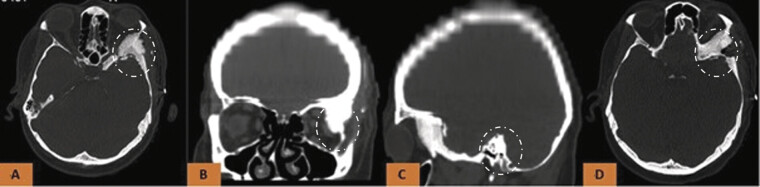
Fibrous dysplasia. An intradiploic, hyperdense, expansile lesion measuring 42 × 32 × 26 mm with a homogeneous ground-glass appearance is noted in the left orbit and zygomatic bone on axial, coronal, and sagittal cranial CT images ( **A** , **B** , **C** ). A postoperative axial cranial CT image shows the biopsy site ( **D** ) (white circle). CT: computed tomography.

A 46-year old woman presented with downward displacement of the left eye, impaired vision, headache and swelling around left eye which led to skull deformation. A biopsy revealed fibrous dysplasia. An intradiploic lesion caused destruction of the left orbital and zygomatic bone, as seen on axial, coronal and sagittal cranial CT scan ( Fig. 8A , Fig. 8B , Fig. 8C ). The site of the biopsy could be seen on postoperative axial cranial CT scan ( Fig. 8D ). The patient was advised to undergo orbital reconstruction. However, the patient declined.

#### Case 9, eosinophilic granuloma

**Fig. 9:**
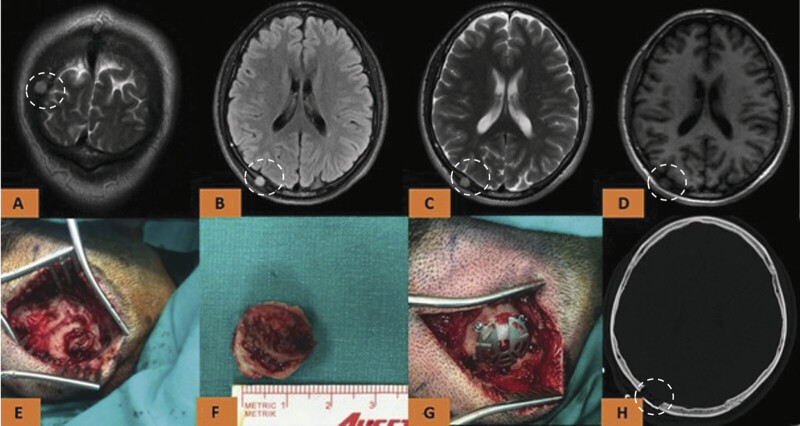
**Right posterior parietal eosinophilic granuloma.** An intradiploic, isointense cystic mass (12 × 11 × 9 mm) lesion on coronal T2-weighted MR images ( **A** ). An intradiploic, hyperintense mass lesion on axial FLAIR MR images ( **B** ). An intradiploic, isohyperintense cystic mass lesion on axial T2-weighted MR images ( **C** ). An intradiploic, isointense mass on axial T1-weighted images ( **D** ). Intraoperative image ( **E** ). Excised intradiploic mass lesion with surrounding normal skull fragments ( **F** ). Burr hole reconstruction ( **G** ). Excision site on postoperative CT scan ( **H** ), (white circle). FLAIR, fluid-attenuated inversion recovery; MR, magnetic resonance.

An 18-year old boy presented with persistent headache following head trauma that occurred 3 months ago. In another hospital, it was reported that a cystic lesion was developed. An intradiploic, isointense cystic mass was seen in the parietal region on coronal T2-weighted MR images ( Fig. 9A ). On axial T1-weighted MR images, the lesion was hyperintense ( Fig. 9B ). On axial T2-weighted MR images, the intradiploic cystic lesion was iso- to hyperintense ( Fig. 9C ). On axial fluid-attenuated inversion recovery (FLAIR) MR images, the lesion was isointense ( Fig. 9D ). The patient underwent surgery. Intraoperative view of the intradiploic mass ( Fig. 9E ). Excised intradiploic lesion ( Fig. 9F ). Reconstruction of the excision site with a titanium burr hole cap. ( Fig. 9G ). The postoperative CT scan showed the excision at the right posterior parietal region. The histopathological examination showed an eosinophilic granuloma. Chemotherapy was initiated after surgery. A control CT revealed no abnormality requiring surgery. The postoperative course was uneventful without an new neurological deficits ( Fig. 9H ).

### Treatment Results

Treatment approaches varied, including total resection with mini-plate reconstruction for the patient with a cavernous hemangioma, total resection for the leptomeningeal cyst ( *n* = 1), and intradiploic lipoma ( *n* = 1). Notably, four patients (44.4%), including those with an epidermoid cyst ( *n* = 2), dermoid cyst ( *n* = 1), and arachnoid cyst ( *n* = 1), were managed conservatively without surgery.

Biopsy was performed for the fibrous dysplasia case ( *n* = 1), and total mass excision with chemotherapy for the eosinophilic granuloma ( *n* = 1).

## Discussion

Intradiploic lesions can originate from various elements of bone, including osteogenic, chondrogenic, fibrogenic, vascular, and other structures. 12 Recognizing the imaging features that distinguish benign from malignant diploic masses is crucial for accurate radiological diagnosis. 2 These lesions can manifest as lytic or sclerotic, single or multiple, each exhibiting distinct characteristics on imaging. 6 Generally, benign tumors present with well-defined borders and a narrow transition zone, often accompanied by sclerotic features. On the other hand, malignant tumors typically exhibit indistinct borders, a broad transitional zone, aggressive periosteal reaction, and may include soft tissue components. 1 Importantly, these lesions have the potential to cause significant bone damage with the possibility of intracranial or extracranial spread. 2

Benign lesions encompass a variety of conditions, including fibrous dysplasia, osteoma, Langerhans cell histiocytosis, venous vascular malformation (cavernous hemangioma), leptomeningeal cyst, eosinophilic granuloma, epidermoid tumor, dermoid tumor, osteoblastoma, aneurysmal bone cyst, meningioma, Paget's disease, and other rare diseases. Malignant lesions that can impact the calvarium include metastases, multiple myeloma, osteosarcoma, chordoma, and chondrosarcoma. Additionally, systemic diseases such as chronic anemia, renal osteodystrophy, and osteopenia have the potential to affect the skull. 9

Intradiploic cysts can become symptomatic by a mass effect or by inducing intracranial hypertension related to epidural or subdural hemorrhage. 13 Consistent with findings in the existing literature, our study showed that intradiploic mass lesions were predominantly located in the parietal and temporal bones. 14 15 16 Notably, our study underscores that surgery may not be universally mandatory for all intradiploic mass lesions.

Table 2 systematically reviews intradiploic lesions reported in the present study and the literature. Demographic and diagnostic data in our series align with those reported in previous studies Comparative analysis reveals that the therapeutic management reported in the literature exhibits less comprehensive details than our series, notably lacking intraoperative and postoperative imaging and detailed histopathological data, particularly in lipoma cases. We believe that our study, by presenting detailed medical, follow-up, and surgical treatment modalities, along with postoperative outcomes, significantly contributes to the existing literature.

**Table 2:** Demographic and clinical features of patients with intradiploic lesions: a literature review

References	Age	Gender	Clinical presentation	Localization	Preop imaging—X-ray	Preop imaging—CT	Preop imaging—MRI	Postoperative imaging	Diagnosis	Management
(Lloret et al 2009)	14 d	F	NA	Parietal, parasagittal	N	N	Y	NA	Atretic cephalocele	NA
	56 y	F	NA	Left frontal	N	Y	Y	NA	Epidermoid cyst	NA
	1 y	M	NA	Parietal, parasagittal	N	Y	Y	NA	Dermoid cyst	NA
	2 y	M	NA	Left parietal	N	Y	Y	NA	Leptomeningeal cyst	NA
	27 y	F	NA	Left parietal	N	Y	Y	NA	Eosinophilic granuloma	NA
	36 y	M	NA	Left frontal	N	Y	Y	NA	Osseous hemangioma	NA
	20 y	M	NA	Left frontal	N	Y	Y	NA	Fibrous dysplasia	NA
	48 y	F	NA	Left orbitozygomatic	N	Y	Y	NA	Meningioma	NA
	38 y	M	NA	Right parietal	N	Y	Y	NA	Osteosarcoma	NA
	84 y	F	NA	Left parietal	N	Y	Y	NA	Angiosarcoma	NA
(Colas et al 2015)	75 y	F	NA	Parietal, parasagittal	N	Y	Y	NA	Metastasis of breast cancer	NA
	8 y	F	NA	Left frontoparietal	Y	Y	N	NA	Langerhans histiocytosis	NA
	52 y	F	NA	Left parietal, parasagittal	N	Y	Y	NA	Intraosseous meningioma	NA
	47 y	F	NA	Left parietal	N	N	Y	NA	Osseous hemangioma	NA
	26 y	F	NA	Right parietal	N	N	Y	NA	Epidermoid cyst	NA
	10 y	M	NA	Parietal, parasagittal	N	N	Y	NA	Dermoid cyst	NA
	30 y	M	NA	Frontal, parasagittal	N	N	Y	NA	Aneurysmal bone cyst	NA
	62 y	M	NA	Left frontal	N	Y	Y	NA	Fibrous dysplasia	NA
(Garfinkle et al 2011)	21 y	M	NA	Left frontal	Y	Y	Y	CTI	Langerhans histiocytosis	Excision
	78 y	F	NA	Parietal, parasagittal	N	Y	N	NA	Epidermoid cyst	NA
	32 y	M	NA	Left orbitofrontal	N	N	Y	NA	Dermoid cyst	NA
	30 y	F	Headache, blurred vision, right proptosis	Right frontal	Y	Y	Y	NA	Hemangioma	NA
	43 y	F	NA	Parietal, parasagittal	N	Y	Y	NA	En plaque meningioma	NA
	48 y	F	NA	Left occipital	N	Y	Y	NA	Hemangiopericytoma	NA
	27 y	F	NA	Right frontal	Y	Y	Y	NA	Fibrous dysplasia	NA
	68 y	F	NA	Right temporal	Y	Y	N	NA	Osteoma	NA
	18 y	F	NA	Left frontal	N	Y	Y	NA	Osteoblastic osteosarcoma	NA
(Arana and Martí-Bonmatí 1999)	27 y	F	tenderness in occipital area	Right occipital	N	Y	N	NA	Eosinophilic granuloma	NA
	17 y	F	Bone swelling	Left frontal	N	Y	Y	NA	Eosinophilic granuloma	Surgical excision
	63 y	F	NA	Left frontal	N	Y	Y	NA	Epidermoid cyst	NA
	43 y	M	NA	Occipital, midline	N	Y	N	NA	Dermoid cyst	NA
	61 y	F	Gait disorder	Right frontal	N	Y	Y	NA	Meningioma	NA
	65 y	M	Headache, palpable mass	Left frontal	N	Y	Y	NA	Meningioma	NA
	47 y	M	Palpable mass	Left parietal	N	Y	Y	NA	Intraosseous meningioma	NA
	42 y	F	Palpable mass	Left parietal	N	Y	Y	NA	Hemangioma	NA
	28 y	F	NA	Right frontal	N	Y	N	NA	Fibrous dysplasia	NA
	32 y	F	Palpable mass	Left occipital, parasagittal	N	Y	Y	NA	Fibrous dysplasia	NA
(Yim et al 2016)	4 y	F	NA	Right frontal	Y	Y	Y	NA	Langerhans histiocytosis	NA
	45 y	M	NA	Right parietal, parasagittal	Y	N	Y	NA	Osseous hemangioma	NA
	13 y	M	NA	Right parietal	Y	Y	Y	NA	Epidermoid cyst	NA
	58 y	F	NA	Right parietal	Y	Y	Y	NA	Intraosseous meningioma	NA
	35 y	M	NA	Occipital, midline	N	Y	Y	NA	Fibrous dysplasia	NA
	1 y	F	NA	Right frontoparietal	N	Y	Y	NA	Desmoplastic fibroma	NA
	45 y	M	NA	Parietal, parasagittal	Y	N	Y	NA	Atretic cephalocele and persistent falcine sinus	NA
(Yalcin et al 2007)	19 y	F	NA	Right frontal	Y	Y	Y	NA	Eosinophilic granuloma	NA
	63 y	M	NA	Left parietal	N	Y	Y	NA	Leptomeningeal cyst	NA
	24 y	F	NA	Right temporal	Y	Y	Y	NA	Fibrous dysplasia	NA
	45 y	F	NA	Left frontoparietal	N	Y	N	NA	Sclerotic intraosseous meningioma	NA
	28 y	F	NA	Right orbitozygomatic	N	Y	N	NA	Osteosarcoma	NA
(Khodarahmi et al 2021)	24 y	M	Seizures, history of fall at 3 y of age	Left parietotemporal	Y	N	Y	NA	Leptomeningeal cyst	NA
	9 mo	F	NA	Parietal, parasagittal	N	Y	N	NA	Epidermoid cyst	NA
	4 y	M	NA	Parietal, parasagittal	N	N	Y	NA	Atretic cephalocele	NA
	18 y	M	NA	Frontoparietal	Y	N	N	NA	Dermoid cyst	NA
	55 y	M	NA	Right parietal	Y	N	Y	NA	Fibrous dysplasia	NA
	29 y	M	NA	Right frontal	N	Y	N	NA	Fibrous dysplasia	NA
	50 y	F	NA	Left orbitozygomatic	N	Y	N	NA	Primary intraosseous meningioma	NA
	52 y	M	NA	Frontal, midline	Y	Y	Y	NA	Hemangioma	NA
	11 y	F	Painful bump on forehead	Left frontal	Y	Y	Y	NA	Langerhans histiocytosis	NA

Abbreviations: CT, computed tomography; F, female; M, male; MRI, magnetic resonance imaging; N, no; NA, not available; Preop, preoperative; Y, yes.

## Cavernous Hemangioma

Calvarial cavernous hemangiomas represent a rare subset of osseous neoplasms, accounting for approximately 2% of all bone tumors. 17 Calvarial hemangiomas are predominantly classified as cavernous on a histological level, characterized by dilated blood vessels situated within the bone trabeculae. 18 This benign and slow-growing vascular bone tumor constitutes less than 1% of primary bone lesions and approximately 10% of benign calvarial tumors. 2

CT features include an intradiploic expansile lesion with trabeculations and spicules. Peripheral sclerosis may also be noted in some cases. There occurs expansion of outer table in most of the cases with sparing of inner table of the skull. 19

After the injection of contrast material, MRI depicts a contrast enhancement which is initially focal and later diffuses. Osseous hemangioma appears iso- to hypointense on T1-weighted MR images and hyperintense on T2-weighted and FLAIR MR images. It may exhibit hypo- or hyperintense spots, indicative of the presence of fat or iron. However, imaging is generally nonspecific, and there is the possibility of an aggressive course with soft tissue invasion. 7 14

Treatment is done in all cases unless specifically warranted. The indications for treatment include symptomatic mass effect on underlying brain, hemorrhage, esthetic aspects, and transformation to aggressive form. 20

The primary mode of treatment is typically surgical, with the option of pre-operative embolization. While radiotherapy can halt tumor progression, it does not lead to a reduction in tumor volume. 21 Embolization and intralesional steroid injection techniques have also been tried to treat the calvarial hemangiomas. 22 In our patient, the cavernous hemangioma was excised and mini-plate reconstruction was performed.

## Leptomeningeal Cyst

Leptomeningeal cysts are predominantly observed in pediatric age groups and often arise as a complication following trauma. Typically, they manifest in the frontoparietal bone after calvarial fractures in children under 3 years of age. The development of leptomeningeal cysts is associated with the enlargement of fractures and the formation of cysts due to CSF pulsation. 23 However, post-traumatic leptomeningeal cysts can occur at any age.

On T1-weighted MR images, these cysts appear hypointense, whereas on T2-weighted MR images, they present as hyperintense. 24 Arachnoid cysts can be identified on CT imaging, exhibiting a hypodense intradiploic lesion with a normal CSF appearance. To differentiate these lesions from dermoid and epidermoid cysts, the addition of MRI is recommended. 25 Typically, these cysts do not necessitate treatment unless becoming symptomatic. 26 In our case, the leptomeningeal cyst, which caused headache and swelling in the parietal area, was surgically excised.

## Intradiploic Lipoma

Intradiploic lipomas can manifest across a broad age range, spanning from 5 to 85 years, with the most common age of discovery typically being between the 4 ^th^ and 5 ^th^ decades of life. There is a slight male predominance, with a male-to-female ratio of approximately 1.3 to 1. 27 The primary symptom in 70% of cases is pain accompanied by palpable soft swelling of the skull. Interestingly, over 30% of intradiploic lipomas are incidentally discovered during imaging studies for other reasons. Histopathological examination reveals lipid vacuoles, and in the literature, intradiploic angiolipoma is frequently reported. On CT imaging, angiolipoma appears nonhomogeneous and hypodense. 28 Our series included one patient with an intradiploic lipoma. In this case, the lipoma was identified as a homogeneous and hyperdense lesion on CT. Cranial MRI revealed homogeneity and hypointensity on T1, and hyperintensity on T2 and FLAIR MR sequences. Generally, surgical intervention is not recommended unless intradiploic lipomas cause symptoms. 29 Our patient underwent lipoma resection due to prominent headaches.

## Epidermoid Cyst

Epidermoid cysts, rare congenital lesions originating from the ectoderm, account for 0.3 to 1.8% of all intracranial tumors. 30 These cysts are believed to develop due to intradiploic congenital factors or included epidermal or dermal particles after trauma. The primary manifestation is typically painless swelling of the scalp. Epidermoid cysts, lined with thin squamous epithelium, may harbor deposits of cholesterol and keratin. 31 Notably, the occurrence of epidermoid cysts is reported to be one times higher than that of dermoids. 13 32 Intradiploic epidermoid cysts are rare, benign, and slow-growing tumors localized in the intertabular space of the cranial bones. They seldom necessitate surgical treatment. 33

On CT scans, epidermoid cysts manifest as well-defined osteolytic lesions with a sclerotic wall, demonstrating a tendency to expand into both the inner and outer layers. They present as homogeneous and hypodense on CT images.

In MRI, these cysts are isointense on T1 MR images. On FLAIR MR sequences, the lesion, which is rich in keratin and protein appears hyperintense. 33 . The lesion is hyperintense and heterogeneous on T2-weighted images and show high-signal intensity on diffusion-weighted imaging (DWI). <Typically, there is no contrast enhancement; however, secondary contrast enhancement may be observed on CT and MRI, attributed to inflammation. 34 35

Notably, epidermoid cysts do not exhibit contrast enhancement;

The lesions of the two patients of the present series exhibited hyperintensity compared with CSF on FLAIR sequences. One patient in our study presented with both an intradiploic epidermoid cyst and a meningioma simultaneously, which underscores the possibility of intradiploic mass lesions coexisting with other intracranial lesions. The other patient was diagnosed with an epidermoid cyst and a subependymal giant cell astrocytoma on cranial MRI. 36 Despite the absence of clinical findings suggestive of tuberous sclerosis, a close follow-up without surgery was planned. Malignant transformation is rare in epidermoid cysts, with a few reported cases of squamous cell carcinoma originating from primary intradiploic epidermoid cysts. 37 38 Surgical resection is typically undertaken to alleviate the mass effect, prevent abscess formation, and mitigate potential complications such as bleeding and malignant transformation. 39 40 41 In our series, as no radiological features suggestive of malignant transformation were observed and the patients were managed without surgery.

## Dermoid Cyst

Dermoid cysts are encapsulated by a thick epithelium and typically contain sebaceous and apocrine sweat glands, and occasionally, hair or teeth. These cysts are commonly detected in childhood or young adults, often appearing along the midline, particularly near the anterior fontanelle. 42

On CT, a dermoid cyst manifests as a hypodense, nonenhancing intradiploic mass lesion. 43 Notably, the presence of fatty signal intensity on T1-weighted images and the prominence of a thick peripheral capsule after gadolinium administration can be crucial findings. 1 In our study, the patient with a dermoid cyst displayed hyperintensity on T1 and a nonhomogeneous isointense appearance on T2, aligning with findings in the existing literature.

Dermoid cysts have the potential to harbor remnants of hair, nails, and skin. Complications often arise due to cyst rupture. 44 However, the precise pathophysiology behind cyst rupture remains uncertain. Rupture may occur spontaneously or as a result of trauma. 45 Similar to dermoid cysts in other body regions, 46 the cyst size and the potential for rupture and infection should be considered during surgical intervention. Our patient underwent surgery tailored to the specific characteristics and dimensions of the cyst.

## Arachnoid Cyst

The occurrence of arachnoid cysts, typically benign pathologies, are within the intradiploic area is rare, and the term “intradiploic arachnoid cyst” was first coined by Weinand et al. 47 These cysts arise from small defects in the dura mater, eroding the inner layer of the folded extensions of the arachnoid membrane. They expand into the diploe, causing damage to the outer layer of the skull. Dunkser and McCreary termed these cysts, “leptomeningeal cysts.” 48 Originating from the inner surface of the affected bone, these cysts may lead to the rupture of the dura, resulting in the accumulation of CSF in the diploic space covered with arachnoid membrane. It is important to note that CSF accumulation in the diploic space is exceedingly rare. 26 49

In the MRI of one of our patients, the arachnoid cyst appeared hypointense in T1-weighted diffusion-weighted imaging and hyperintense in T2-weighted Diffusion-Weighted Imaging. The patient is currently being monitored and surgery is not being considered.

## Fibrous Dysplasia

Fibrous dysplasia is an exceptionally rare bone disease typically occurring in the first 20 years of life. 50 51 Resulting from a nonhereditary genetic mutation, this condition leads to abnormal differentiation and maturation of osteoblasts, causing the progressive replacement of normal bone with immature woven bone. Bone involvement can be focal or multifocal, with a hemicranial predominance when extensive. The frontal bone is most commonly affected, followed by the sphenoid, ethmoidal, parietal, temporal, and occipital bones. 50 Fibrous dysplasia can lead to expansion and distortion of the affected bones. 52 Although fibrous dysplasia is rare in the craniofacial region 53 and is not typically classified as an intradiploic mass, it was included in this study due to its involvement of the bone structure in the present case.

For diagnosis, CT is the preferred imaging modality, capable of revealing the characteristic ground-glass appearance (70–130 HU) in all or part of the lesion. 54 Intralesional calcifications may also be present. Medical treatment often involves the use of bisphosphonates. Surgical decompression is considered in cases with a severe mass effect. 12 In our study, the patient with fibrous dysplasia underwent clinical follow-up after biopsy.

## Eosinophilic Granuloma

Langerhans cell histiocytosis (LCH) encompasses three idiopathic diseases characterized by the proliferation of Langerhans cells. 55 When the lesion is solitary and monostotic, as is often the case, it is termed eosinophilic granuloma. 56 LCH is most prevalent in children and adolescents, and its severity tends to be inversely proportional to the age of onset. 57 The skull is the most common site affected by LCH, with the parietal bones being the most commonly involved. 58 Typically, the lesion is well-defined, lytic with nonsclerotic borders, and it extends across all three layers of the skull. 59

Eosinophilic granuloma is a subtype of LCH, constituting a rare entity that accounts for less than 1% of all bone tumors. 60 It is more commonly found in the calvarium, mandible, ribs, ilium, or long bones, presenting with symptoms such as swelling, deformation, and tenderness. 56 In cases where there is definite indication for surgery, close follow-up is recommended, as these lesions may become symptomatic (e.g., hematoma due to cyst perforation or bleeding). 59 In our study, the patient with eosinophilic granuloma in the left parietal area underwent chemotherapy after mass excision due to symptomatic headaches.

## Limitations

This study has several limitations. First, the generalizability of our findings is constrained by the limited sample size and the heterogeneity of diagnoses within our patient cohort. However, it is important to emphasize that intradiploic masses are rare entities, which inherently poses challenges to perform larger, comparative studies. Second, the absence of systematic, long-term clinical and imaging follow-up represents a notable shortcoming. As some lesions had been asymptomatic, routine follow-up imaging was not uniformly pursued, and subsequent evaluations were primarily guided by clinical presentation. On the other hand, this study has several strengths. Notably, it provides detailed radiological and surgical characterization of rare disease subtypes. Furthermore, the expert neuroradiological interpretations presented herein contribute valuable insights.

Due to the relative rarity of benign lesions of the skull and the similarity of some imaging findings, they are easily confused with one another or misdiagnosed. It is important to understand the imaging characteristics of these diseases to make an accurate diagnosis based on a patient's clinical history and laboratory tests. 61 62

## Conclusions

Intradiploic lesions are uncommon entities, posing diagnostic and therapeutic challenges. While CT and MRI are valuable tools for characterizing these masses, achieving a definitive diagnosis often necessitates histopathological evaluation. 28 33 Surgical excision, followed by thorough pathological examination, remains the gold standard for establishing a conclusive diagnosis. Current best practices emphasize a tailored approach to management, considering the patient's unique radiological and clinical presentation. In cases where surgery is deemed necessary, complete excision is the ideal treatment. However, for asymptomatic patients or those with lesions suggestive of benign etiology, a conservative approach involving close clinical and radiological monitoring may be warranted. Ultimately, a multidisciplinary assessment, integrating radiological findings, clinical symptoms, and surgical expertise, is crucial for optimizing patient outcomes.
